# Exploring the Impact of Muscle Vibration Therapy in Neurologic Rehabilitation: A Systematic Review

**DOI:** 10.1016/j.arrct.2025.100478

**Published:** 2025-06-06

**Authors:** Andrea Calderone, Svonko Galasso, Alessandro Marco De Nunzio, Antonio Leo, Tina Balletta, Angelo Quartarone, Rocco Salvatore Calabrò

**Affiliations:** a Department of Clinical and Experimental Medicine, University of Messina, Messina, Italy.; b Department of Health, LUNEX University of Applied Sciences, Differdange, Luxembourg.; c Luxembourg Health & Sport Sciences Research Institute A.s.b.l., Differdange, Luxembourg.; d Department of Electrical and Information Engineering, University of Cassino and Southern Lazio, Cassino, Italy.; e IRCCS Centro Neurolesi Bonino-Pulejo, Messina, Italy.

**Keywords:** Motor function improvement, Muscle vibration, Neurologic disorders, Neuroplasticity, Neurorehabilitation, Rehabilitation, Therapeutic interventions

## Abstract

**Objective:**

To consolidate evidence on the efficacy of muscle vibration therapy for neurorehabilitation, providing health care practitioners with insights for enhancing treatment protocols and guiding future research.

**Data Sources:**

Studies were identified from an online search of PubMed, Web of Science, and Embase databases, with a search time range of 2014-2024.

**Study Selection:**

A total of 26 studies involving 787 individuals were included in this systematic review, including diverse neurologic conditions and intervention protocols.

**Data Extraction:**

Keywords, Boolean operators, and controlled vocabulary were combined and tested in a gradual and iterative manner to achieve the highest possible sensitivity and specificity. The PRISMA flowchart was used to depict the process of selecting relevant studies.

**Data Synthesis:**

Research on segmental and local muscle vibration in upper limb rehabilitation for poststroke patients is promising, as it can improve motor function, decrease spasticity, and enhance muscle control. Whole-body vibration interventions also show advantages in lower limb spasticity and balance, with specific studies adducing better results when paired with task-specific training. Vibration therapy has shown promising outcomes for alleviating pain, managing spasticity, and improving motor function in various neurologic conditions such as SCI and cerebral palsy, highlighting its potential in treating different neurologic disorders.

**Conclusions:**

This review emphasizes the potential of muscle vibration therapy in neurorehabilitation, showing benefits in motor control, spasticity, and functional outcomes, while underscoring the importance of rigorous methods and further extensive research to improve result dependability.

Neurologic disorders represent a significant global health challenge, affecting millions and contributing to death and disability, particularly in the elderly. According to the World Health Organization, these disorders account for 6.3% of the global illness burden, with stroke and dementia among the most debilitating and fatal conditions.^1^, ^2^, ^3^, ^4^, ^5^, ^6^, ^7^ Rehabilitation is crucial for restoring functional abilities and improving quality of life.^8^^,^^9^ Muscle vibration therapy (MVT) has gained attention as a promising noninvasive treatment for neurologic conditions. It has been increasingly recognized as a valuable addition to managing disorders such as stroke, multiple sclerosis (MS), spinal cord injury (SCI), cerebral palsy (Cpal), and Parkinson disease (PD).^10^, ^11^, ^12^, ^13^, ^14^, ^15^, ^16^, ^17^, ^18^, ^19^, ^20^, ^21^, ^22^, ^23^, ^24^, ^25^ MVT delivers mechanical vibrations to the whole-body or specific muscles, activating sensory receptors to enhance stimulation. This process improves coordination and strength, which are essential for motor recovery, by boosting proprioceptive feedback.^26^ Research has demonstrated that MVT improves grip strength, movement control, balance, and posture stability, reducing fall risks in patients with neurologic disorders, including stroke survivors seeking to regain complex motor skills.^27^, ^28^, ^29^ The noninvasive nature and compatibility of MVT with other interventions make it a versatile addition to personalized rehabilitation programs, thereby enhancing outcomes.^30^ While preliminary research highlights the potential of MVT, consensus on its optimal use, effectiveness, and mechanisms is lacking. In addition to its peripheral effects, MVT stimulates the central nervous system.^31^ Studies using functional magnetic resonance imaging and electroencephalography have revealed that MVT activates brain regions linked to motor and sensory functions, enhancing cortical excitability and potentially improving neuroplasticity, the brain’s ability to reorganize and form new connections.^32^, ^33^, ^34^, ^35^, ^36^, ^37^ This is critical for neurorehabilitation aimed at recovering lost motor function. Studies have shown that prolonged vibration therapy induces lasting changes in muscle fiber composition, motor neuron activation, and synaptic efficiency.^38^^,^^39^ These adaptations may enhance motor skills and reduce disabilities in individuals with neurologic disorders. Additionally, neurophysiologic mechanisms highlight the crucial role of neurotransmitters such as GABA and glutamate in the effects of MVs. Specifically, GABA, an inhibitory neurotransmitter, modulates the excitability of spinal cord neurons, potentially reducing excessive muscle tone and spasticity. Glutamate, an excitatory neurotransmitter, enhances synaptic transmission and plasticity, facilitating motor learning and recovery by strengthening neural pathways involved in muscle control.^40^, ^41^, ^42^, ^43^

Despite the evidence highlighted, the current body of literature on the efficacy of MVT in various neurologic conditions is fragmented and lacks a comprehensive synthesis. Specifically, there is a notable gap in the literature regarding a systematic, evidence-based overview of the effectiveness of MVT across the broad spectrum of neurologic disorders. Although individual studies and smaller reviews exist, they often focus on specific conditions or limited aspects of MVT applications. This piecemeal approach makes it difficult for health care practitioners to make informed, evidence-based decisions about MVT integration into clinical practice. Therefore, this systematic review is necessary to address this critical gap by providing a consolidated and up-to-date analysis of the effectiveness of MVT across various neurologic disorders. By synthesizing the existing evidence, this review offers a clear understanding of the potential benefits of MVT, identifies optimal application parameters, and highlights areas for further research. This will empower health care practitioners with the necessary information to confidently and effectively use MVT as a therapeutic modality, ultimately improving patient outcomes and quality of life. In essence, this review will serve as a crucial resource, bridging the gap between fragmented research and practical clinical application, thereby facilitating the translation of scientific evidence into improved patient care.

## Theory and calculations

The theoretical basis for using muscle vibration (MV) in neurologic rehabilitation is based on its interaction with the neuromuscular and sensory systems, providing a means of improving motor control and neuroplasticity.^10^ Vibration applied to muscle groups triggers muscle spindles and stimulates proprioceptors, producing signals that travel to the central nervous system through sensory pathways.^44^ This sensory stimulation can affect motor pathways by increasing motor neuron activity, potentially leading to better motor coordination. Moreover, consistent vibration can support beneficial brain alterations, thereby encouraging neuroplasticity, which is a crucial mechanism for enhancing the functioning of individuals with neurologic deficits.^45^ Additionally, MV has been linked to a brief rise in blood circulation and enhanced metabolic function in specific muscle regions, potentially aiding muscle recovery and neuromuscular efficiency.^46^ The vibrating movement causes oscillation that can affect soft tissue, decrease muscle stiffness, and enhance flexibility, which is especially advantageous for patients with spasticity or muscle tightness-related conditions. Specific quantitative factors, including frequency, amplitude, duration, and targeted areas, are crucial for maximizing treatment effectiveness in the calculation part.^47^ Frequencies fall within the 30-100 Hz range, and altering these frequencies can induce diverse neuromuscular reactions.^48^ In the same way, muscle activation and endurance levels can be affected by the intensity and length of the vibration. If not appropriately controlled, prolonged duration may lead to fatigue.^49^ These findings provide a foundation for improving MVT as a structured, evidence-based intervention, providing health care professionals and researchers with a basis for creating specific protocols to enhance motor function and recovery in patients with neurologic disorders.

## Methods

### Inclusion criteria

Studies that met the criteria had to discuss or investigate the use of MVT for people with different neurologic disorders, such as stroke, MS, CPal, diabetic peripheral neuropathy (DPN), spinal cord injuries, and PD. Only English articles were considered to make the analysis consistent. Furthermore, research focusing on evaluating the functionality of individuals receiving MVT was considered, with a focus on functional results to gain a deeper understanding of recovery. The standards were updated to concentrate only on human populations, excluding research involving animals. Participants ranged from young individuals (5-17y) to adults aged ≥18 years who have been diagnosed with one or more of the designated neurologic disorders. More specifically, the criteria for inclusion consisted of original research articles or clinical trial protocols that involved randomized controlled trials (RCTs), uncontrolled experimental studies, comparative studies, retrospective studies, prospective studies, cohort studies, and case-control studies, which also provided detailed information on the methodology and results regarding MVT.

### Exclusion criteria

Specific studies were excluded from the review to maintain the quality and relevance of the included literature. Studies lacking sufficient data or information on the use of MVT for neurologic disorders were deemed unsuitable, particularly those without precise results, methods, or pertinent discussions. Additionally, articles published in languages other than English were excluded to ensure that the review team could effectively evaluate and analyze the studies without language barriers, facilitating a more standardized evaluation process. Systematic, integrated, or narrative reviews were also excluded; however, their reference lists were reviewed and included when relevant.

### PICO evaluation

We applied the PICO model (Population, Intervention, Comparison, Outcome) to create the search terms. The population of this review includes those who have been diagnosed with various forms of neurologic conditions, such as recovering stroke patients, and those affected by MS, CPal, PD, DPN, and spinal cord injuries. The intervention investigated was MVT, which involves the application of mechanical vibrations to a specific muscle or muscles. This review covers different protocols of MV, such as frequency and duration, and their modes of application, such as localized versus whole-body vibration (WBV). The comparison categories included groups that received traditional rehabilitative therapies, sham vibration treatments, and no intervention. The assessment then established whether MV was effective in neurologic rehabilitation by reviewing the range of treatments for such cases, especially when assessing patients’ motor functions, muscle strength and coordination, spasticity assessments, balance, mobility, and overall quality of life measures. The outcomes included physiological measurements, such as blood flow and muscle activation patterns. In examining these results, this study tests whether MV can bring statistically meaningful improvements over control conditions.

### Search strategy

The investigation included available research period of 2014-2024 to comprehensively gather recent knowledge on the efficacy of MVT in various neurologic conditions, ensuring that no relevant recent evidence was missed (time search conduction from October 1-25, 2024). We performed an extensive literature search using the PubMed, Web of Science, and Embase databases, using the keywords: ("muscles"[All Fields] OR "muscles"[MeSH Terms] OR "muscles"[All Fields] OR "muscle"[All Fields]) AND ("vibrate"[All Fields] OR "vibrated"[All Fields] OR "vibrates"[All Fields] OR "vibrating"[All Fields] OR "vibration"[MeSH Terms] OR "vibration"[All Fields] OR "vibrations"[All Fields] OR "vibrational"[All Fields] OR "vibrator"[All Fields] OR "vibrators"[All Fields]) AND ("nervous system diseases"[MeSH Terms] OR ("nervous"[All Fields] AND "system"[All Fields] AND "diseases"[All Fields]) OR "nervous system diseases"[All Fields] OR ("neurological"[All Fields] AND "disorders"[All Fields]) OR "neurological disorders"[All Fields]) AND ("neurological rehabilitation"[MeSH Terms] OR ("neurological"[All Fields] AND "rehabilitation"[All Fields]) OR "neurological rehabilitation"[All Fields] OR "neurorehabilitation"[All Fields] OR "neurorehabilitative"[All Fields]). These databases were carefully selected to cover the broadest possible range of peer-reviewed literature in the fields relevant to this review. PubMed was selected for its deep representation of biomedical research, particularly its rich indexing of neurologic disorders and rehabilitation research. Web of Science was selected for its multidisciplinary coverage and citation tracking functionality, which allows identification of seminal works in the field. Embase was selected for its long-standing reputation for providing broad coverage of clinical and pharmacologic studies, particularly neurorehabilitation studies. By using these databases, we sought to maximize the robustness and therefore validity of our strategy, reduce the possibility of missing important studies, and allow for the inclusion of high-quality, diverse evidence.

#### Data extraction

**Fig 1:**
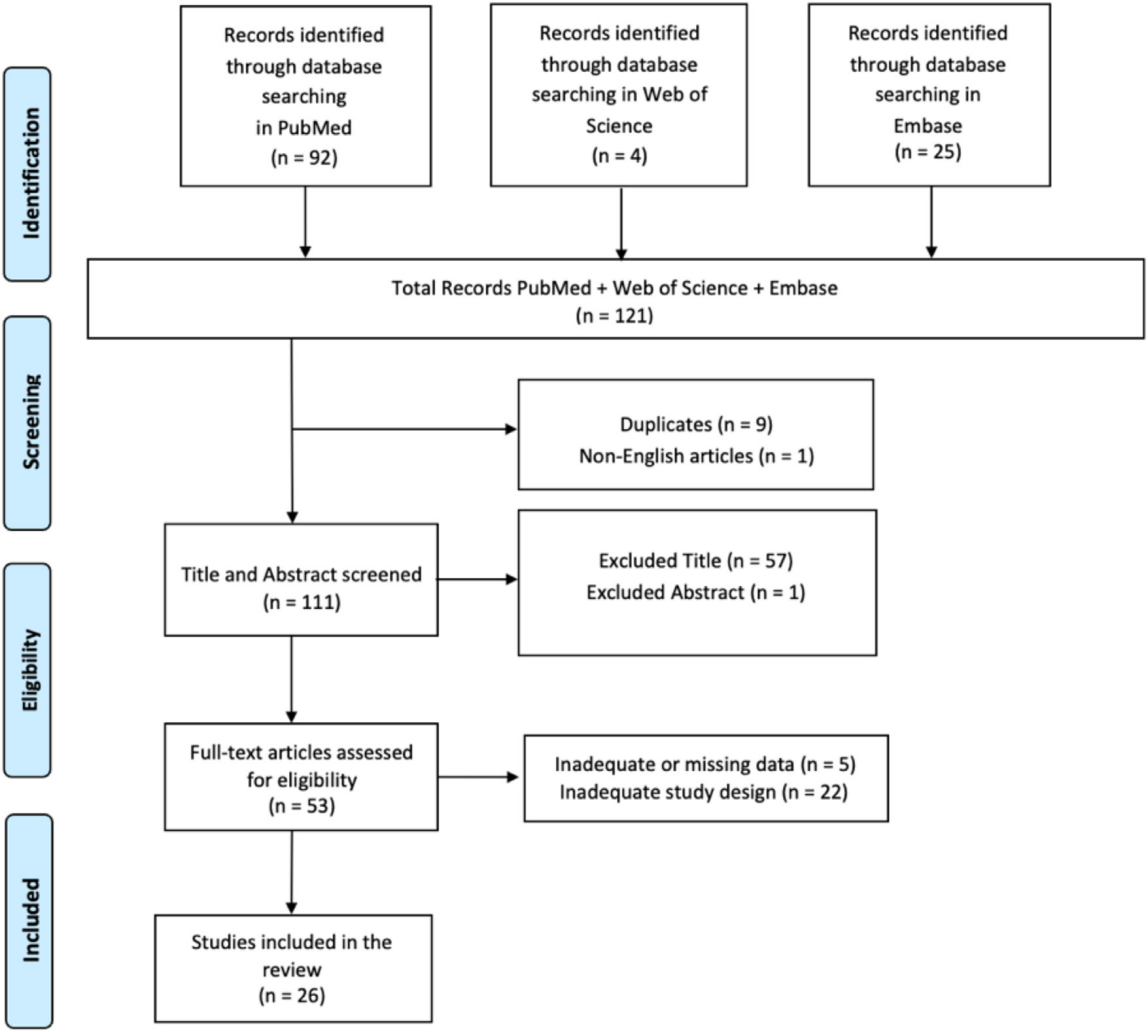
PRISMA 2020 flow diagram of evaluated studies. The diagram illustrates the study selection process, including the number of records identified, screened, excluded, and included in the systematic review. Abbreviation: PRISMA, Preferred Reporting Items for Systematic Reviews and Meta-Analyses.

Two reviewers (A.C., S.G.) performed independent searches to improve transparency and accuracy in locating pertinent studies. The search strategy was iteratively refined by testing different combinations of keywords, Boolean operators, and controlled vocabulary (eg, MeSH terms) to maximize sensitivity and specificity. The PRISMA flowchart was used to depict the process (identification, screening, eligibility, and inclusion) of selecting relevant studies (fig 1).^50^ Additionally, 2 researchers (A.C., S.G.) screened all articles based on titles, abstracts, and full texts, conducting independent data extraction, article gathering, and cross-validation to minimize bias risks (eg, missing results bias, publication bias, time lag bias, language bias). The data gathered included study design, sample size, characteristics of participants, types of neurologic disorders, specifics of the MVT intervention, duration, and assessed outcomes. The researchers (A.C., S.G.) reviewed complete text articles considered suitable for the study, and if there were disagreements regarding the inclusion and exclusion criteria, a final decision was reached by a third researcher (R.S.C.). Discrepancies between reviewers (A.C., S.G.) during the screening or data extraction process were also resolved through discussion, with unresolved cases adjudicated by a third reviewer (R.S.C.). The concordance between the 2 evaluators (A.C. and S.G.) was evaluated using the κ statistic. The κ score, which has a recognized threshold for substantial agreement of >0.61, was understood to indicate substantial alignment among the reviewers.^51^ This standard guarantees a strong assessment of interrater reliability, highlighting the attainment of a significant degree of consensus in the data extraction procedure. Data extraction and organization were facilitated using Microsoft Excel,^a^ which streamlined the process and minimized human error. The software enabled efficient management of large datasets, enabling reviewers to systematically record study characteristics and outcome data. Custom extraction sheets were designed within the software to ensure consistency and adherence to the predefined inclusion/exclusion criteria. In addition, the software provided features such as tagging, filtering, and sorting, which facilitated the resolution of discrepancies and expedited the cross-validation process. This set of articles was subsequently refined for relevance, assessment, and summary, with the main topics highlighted from the summary according to the inclusion/exclusion standards. A detailed protocol for assessing bias, which was aligned with the Cochrane Handbook for Systematic Reviews of Interventions, was followed to maintain methodological rigor. The Cochrane Risk of Bias (RoB 2) framework^b^ was applied to assess bias risk in RCTs, whereas the ROBINS-I tool was used for uncontrolled experimental studies included in this review.

This systematic review has been registered in PROSPERO under the following number: CRD42024605909. Registration on PROSPERO ensures transparency and accountability by providing a publicly accessible record of the review protocol before its execution. This step minimizes the risk of selective reporting and allows external validation of the review methodology. By adhering to PROSPERO standards, this review aligns with best practices for systematic reviews and reinforces its methodological rigor.

#### Data synthesis

Data synthesis was conducted using narrative methods and quantitative analysis to address the variety of study designs and types of neurologic conditions. This method allowed us to identify key themes, commonalities, and differences across the research landscape. By synthesizing qualitative observations, we provided a comprehensive understanding of the evidence related to different populations with various neurologic conditions. Effect sizes and evidence certainty from diverse study types reflecting various neurologic conditions were reported. Studies were grouped according to intervention types, patient characteristics, and reported outcomes to highlight consistencies and discrepancies. Throughout the synthesis process, the inclusion of a multidisciplinary team ensured a balanced interpretation of the data. Regular discussions and consensus meetings among reviewers helped mitigate potential biases in qualitative assessments and ensured consistency in the categorization and interpretation of outcomes. This integrated approach combined qualitative insights with quantitative precision, providing a holistic understanding of the research landscape.

## Results

### Quality of included studies: risk of bias

**Fig 2:**
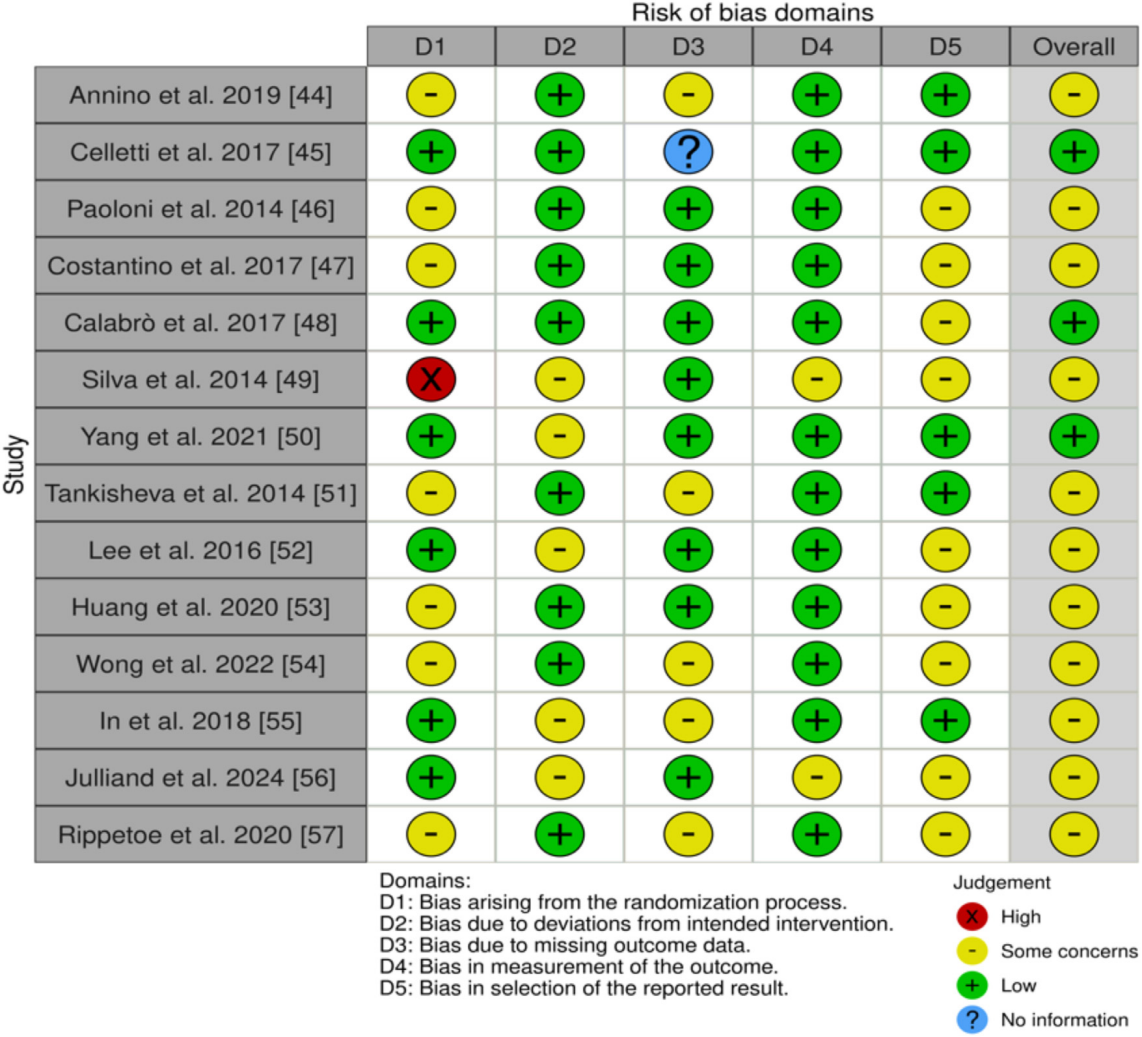
Risk of Bias (RoB) assessment for included randomized controlled trials (RCTs). This figure summarizes the risk of bias across 5 domains (D1-D5) for included RCTs. Each domain represents a specific source of bias, and the overall judgment combines domain-specific assessments. Judgments are color-coded as follows: green (+) = low risk, yellow (-) = some concerns, red (×) = high risk, and blue (?) = no information available. Domains: D1 = Bias from the randomization process; D2 = Bias because of deviations from intended interventions; D3 = Bias because of missing outcome data; D4 = Bias in outcome measurement; D5 = Bias in selecting the reported result.

We assessed the risk of bias using appropriate tools based on the study design of the included studies.^22^^,^^52^, ^53^, ^54^, ^55^, ^56^, ^57^, ^58^, ^59^, ^60^, ^61^, ^62^, ^63^, ^64^, ^65^, ^66^, ^67^, ^68^, ^69^, ^70^, ^71^, ^72^, ^73^, ^74^, ^75^, ^76^ Fourteen of the 26 studies were RCTs.^22^^,^^52^, ^53^, ^54^, ^55^, ^56^, ^57^, ^58^, ^59^, ^60^, ^61^, ^62^, ^63^, ^64^ For these purposes, we used the updated RoB 2 tool, which covers 5 domains: (D1) bias arising from the randomization process; (D2) bias because of deviations from the intended intervention; (D3) bias because of missing data on the results; (D4) bias in the measurement of the outcome; and (D5) bias in the selection of the reported result (fig 2).^77^

Our assessment identified concerns about the overall risk of bias, particularly in the selection of reported results (D5). Annino et al,^52^ Paoloni et al,^54^ Costantino et al,^55^ Tankisheva et al,^59^ Huang et al,^61^ Wong et al,^62^ and Rippetoe et al^22^ presented concerns about the randomization process (D1). Silva et al,^57^ Yang et al,^58^ Lee et al,^60^ In et al,^63^ and Julliand et al^64^ presented concerns regarding D2 (deviations from intended interventions). Most studies showed a low risk of outcome measurement (D4) but had concerns about missing data (D3),^22^^,^^52^^,^^59^^,^^62^^,^^63^ except for Celletti et al,^53^ which requires more information. The severe risk of randomization (D1) was noted in Silva et al.^57^ Although many studies have demonstrated reasonable bias control, methodological issues remain, particularly in terms of randomization and reported result discrepancies. Addressing these biases is essential for improving reliability.

**Fig 3:**
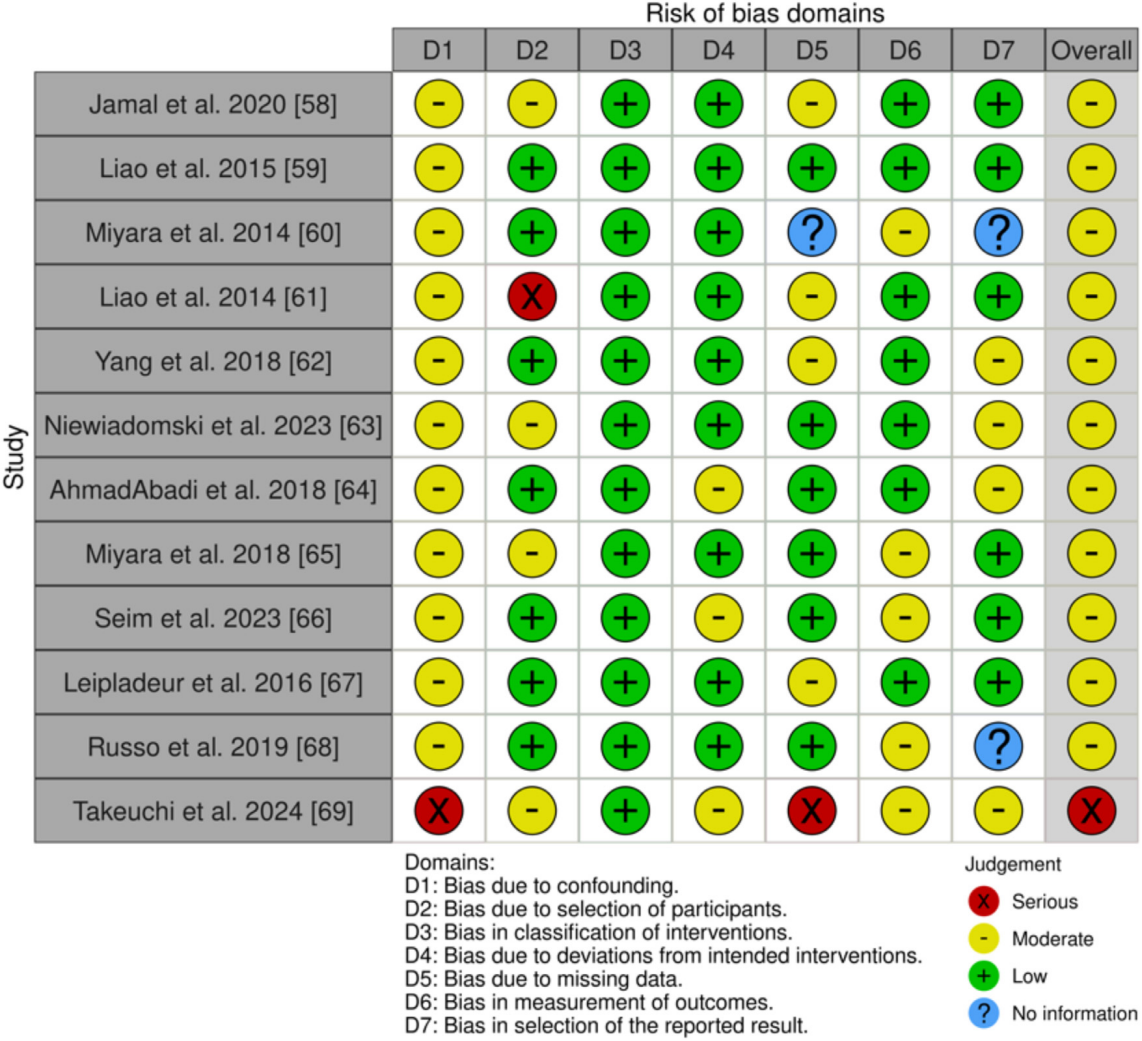
Cochrane Risk of Bias in Nonrandomized Studies of Interventions (ROBINS-I). This figure evaluates the risk of bias across 7 domains (D1-D7) for nonrandomized studies included in the review. Each domain corresponds to specific sources of bias, and the overall judgment combines these assessments. Judgments are color-coded as follows: green (+) = low risk, yellow (-) = moderate risk, red (×) = serious risk, and blue (?) = no information available. Domains: D1 = Bias because of confounding; D2 = Bias in participant selection; D3 = Bias in classification of interventions; D4 = Bias because of deviations from intended interventions; D5 = Bias because of missing data; D6 = Bias in outcome measurement; D7 = Bias in selection of the reported result.

For the 12 nonrandomized studies, 6 uncontrolled experimental studies,^65^, ^66^, ^67^, ^68^, ^69^, ^70^ 2 comparative studies,^71^^,^^72^ 3 prospective studies,^73^, ^74^, ^75^ and 1 retrospective observational study,^76^ we applied the ROBINS-I tool, which assesses 7 areas of bias: (D1) bias because of confounding; (D2) bias in participant selection; (D3) bias in classification of interventions; (D4) bias because of deviations from intended interventions; (D5) bias because of missing data; (D6) bias in outcome measurement; and (D7) bias in the selection of the reported outcome (fig 3).^77^

The ROBINS-I ratings indicate moderate methodological quality, but there are notable concerns. Takeuchi et al^76^ demonstrated a severe risk of bias in D1 (confounding), whereas Liao et al^68^ showed a severe risk in D2 (participant selection). All studies had low risk according to the intervention classification (D3). AhmadAbadi et al,^71^ Seim et al,73 and Takeuchi et al^76^ reported moderate risks in D4 (deviations from interventions), whereas Miyara et al^67^ and Russo et al^75^ needed more information on reported results (D7). Moderate risk in missing data (D5) was noted in Jamal et al,^65^ Liao et al,^68^ Yang et al,^69^ and Leplaideur et al,^74^ and severe risk was noted in Takeuchi et al.^76^ The outcome measurement bias (D6) was generally low^65^^,^^66^^,^^68^, ^69^, ^70^, ^71^^,^^74^ but moderate in Miyara et al,^67^ Miyara et al,^72^ Seim et al,^73^ Russo et al,^75^ and Takeuchi et al.^76^ Although the studies demonstrated adequate methodological quality, moderate to severe biases hinder dependability. Future research must address these limitations to improve evidence-based practices.

### Synthesis of evidence

A total of 121 articles were identified, with 9 removed because of duplication, 1 excluded for not being published in English, and 58 excluded after title and abstract screening. An additional 27 articles were excluded because of inadequate or untraceable study designs (see fig 1). Ultimately, 26 research articles met the inclusion criteria and are summarized in supplemental table S1.^10^^,^^22^^,^^44^, ^45^, ^46^, ^47^, ^48^, ^49^, ^50^, ^51^, ^52^, ^53^, ^54^, ^55^, ^56^, ^57^, ^58^, ^59^, ^60^, ^61^, ^62^, ^63^, ^64^, ^65^, ^66^, ^67^

These studies analyzed data from 787 individuals with various neurologic conditions and intervention types, involving 15-80 participants per study, most with <50 participants. Participants’ ages ranged 20-85 years, with research showing the following distributions: 40-85 years, 50-80 years, 55-75 years, and ≥18 years. The mean age, where provided, ranged from 46.1±9.8 to 61.59±15.50 years. Sex representation varied, with studies including both men and women. Where specific numbers were provided, the studies included 2-34 men and 5-21 women. Clinical outcomes primarily focused on enhancing motor function, increasing muscle strength, reducing spasticity, and managing pain. Frequently used assessment tools included the Modified Ashworth Scale, Fugl-Meyer Assessment, Hand Grip Strength Test, Motor Function Measure, and visual analog scale for pain, along with the FIM, goniometry, and EMG for function and muscle activity evaluation.

#### Study interventions, statistical analyses, and certainty of evidence

This review summarized the results of research examining MVTs across various neurologic conditions, including intervention protocols. In particular, research has examined the use of MVT to improve upper limb function after stroke, with interventions varying from segmental muscle vibration (SMV) combined with conventional physiotherapy (CP)^52^, ^53^, ^54^, ^55^, ^56^^,^^73^^,^^75^ to focal muscle vibration (FMV) along with robotic arm training.^56^ Additional interventions involve using modified wearable devices that deliver functional MV to improve walking patterns in individuals with diabetes and DPN.^22^ Additional research examined the application of WBV to lessen spasticity and enhance motor control in stroke survivors, using protocols that differed in frequency, amplitude, and vibration duration.^57^, ^58^, ^59^, ^60^, ^61^^,^^64^^,^^66^, ^67^, ^68^^,^^71^^,^^72^ Moreover, studies investigated how WBV influences spasticity, postural sway, and gait in individuals with incomplete SCI,^62^^,^^63^ along with the effects of various WBV doses on pain and reflex excitability in those experiencing chronic neuropathic pain.^62^ Interventions also involved employing noninvasive MV to evaluate postural control and balance in chronic stroke patients with right- and left-brain injury.^74^ The variation in treatment protocols highlights the necessity for an in-depth examination of the statistical techniques used to evaluate the effectiveness of these interventions. Statistical analyses predominantly employed nonparametric tests like the Mann-Whitney *U*, Wilcoxon signed-rank, and *t* tests, with adjustments such as Bonferroni correction. Effect sizes, which were often measured using Cohen’s *d*, revealed moderate to significant interventions. Evidence certainty was generally moderate because of sample size constraints, study design limitations, and outcome variability, although some rigorously designed studies indicated high certainty.

Key findings about MVT were categorized by condition: 7 studies investigated segmental and localized vibration on upper limb movement in poststroke patients^52^, ^53^, ^54^, ^55^, ^56^^,^^73^^,^^75^; 11 explored WBV effects on spasticity and motor control in poststroke individuals^57^, ^58^, ^59^, ^60^, ^61^^,^^64^^,^^66^, ^67^, ^68^^,^^71^^,^^72^; 3 examined vibration therapy for SCI and CPal^62^^,^^63^^,^^75^; and 5 analyzed FMV and WBV effects on gait and stability in neurologic disorders.^22^^,^^65^^,^^69^^,^^70^^,^^74^ A qualitative analysis using thematic synthesis identified common themes, trends, and discrepancies in approaches, treatment locations, and patient responses, highlighting the importance of MVT in managing neurologic disorders.

### Effects of segmental and local muscle vibration on upper limb movement poststroke

Numerous studies have examined the effects of SMV and LMV on upper limb function in poststroke hemiparetic individuals. Controlled experiments aimed at improving motor control for specific motions, such as reaching, have shown the benefits of segmental vibration in the upper limb muscles. In this RCT, 37 ischemic stroke patients were split into 2 groups: one receiving supervised physical therapy (SPT) and the other receiving SPT with SMV. After the departure of 3 participants, 34 completed the 8-week program. Both groups experienced improvement in the Barthel Index, elbow range of motion, and muscle strength. Enhanced muscle tone in the affected limb was observed only in the SPT-SMV group, highlighting its specific benefits.^52^ Another study with 18 chronic stroke patients divided participants into 3 groups: one receiving FMV with progressive modular rebalancing, another receiving FMV with traditional physiotherapy, and a control group receiving only traditional physiotherapy. Both FMV groups demonstrated significant improvements in the Wolf Motor Function Test and Motricity Index scores after 6 weeks of treatment, along with reduced pain levels as assessed by the visual analog scale. Spasticity, measured with the Modified Ashworth Scale, also decreased in all groups.^53^ Paoloni et al^54^ explored the effects of low-amplitude SMV in patients with stroke and chronic hemiparesis. Thirty-five individuals were randomly divided into experimental and control groups. The SMV group exhibited significant improvements in muscle activation speeds and decreased cocontraction indices, suggesting better motor coordination and reduced resistance during movement.^54^ LMV therapy further demonstrated the potential to enhance grip strength and daily functionality while reducing pain and spasticity in poststroke patients.^55^ Robotic-assisted rehabilitation combined with genuine vibrotactile feedback (MV) significantly improved neuromuscular activation and clinical outcomes, particularly in motor control and functional autonomy.^56^

A prospective study on the Vibrotactile Stimulation Glove revealed its efficacy in reducing hand spasticity and enhancing active finger extension in chronic stroke patients. The improvements persisted 4 weeks postintervention, suggesting the glove as a potential alternative to botulinum toxin type A treatment.^73^

Additionally, a retrospective observational study compared stretching, tendon vibration, and muscle belly vibration for managing spasticity in 27 stroke patients. Tendon vibration is most effective for reducing finger flexor spasticity, with lasting effects noted for wrist flexors when combined with voluntary movement.^76^

Overall, the findings underscore the therapeutic value of SMV and LMV in improving motor function, reducing pain, and managing spasticity in stroke rehabilitation.

### Effect of whole-body vibration on spasticity and motor control in lower and upper limbs poststroke

Research studies have demonstrated the potential of WBV as an additional treatment for enhancing motor function and managing spasticity in stroke patients. Participants were divided into vibration training and control groups in a RCT involving 43 patients with hemiparesis. Although muscle strength and balance improvements were observed in the vibration group, no significant differences in motor function were noted compared with the control group. However, control group participants showed superior results in the 6-minute walk test and the timed Up and Go test.^57^ Another study evaluated the effects of different WBV frequencies on muscle strength and bone turnover in patients with chronic stroke. Eighty-four participants were randomized into 20-Hz and 30-Hz vibration groups and underwent an 8-week program. The 30-Hz group exhibited significantly improved leg muscle power and reduced bone breakdown markers.^58^ Lee et al^60^ conducted a study with 45 poststroke patients to compare task-specific training combined with WBV to WBV alone and control groups. Patients in the combined training group exhibited significant improvements in arm function, grip strength, and spasticity reduction, highlighting the benefits of integrating WBV with task-specific exercises.^60^ The short-term effects of WBV on spasticity and blood flow were also explored by Hung et al. in a study with 36 patients with chronic stroke. WBV suppressed hyperreflexia and enhanced blood flow in the medial gastrocnemius muscle, thereby improving muscle health.^61^ Research focusing on lower limb spasticity highlighted the benefits of WBV in improving balance and reducing spasticity. Active vibration was more effective than placebo treatment, as evidenced by immediate balance and ankle function enhancements in 22 stroke patients.^71^ Miyara et al^72^ demonstrated that WBV reduced spasticity and increased range of motion, suggesting its potential in regulating muscle excitability. An uncontrolled experimental study involving 25 poststroke patients with lower limb spasticity further reinforced the role of WBV in reducing spasticity and enhancing gait parameters such as walking speed and cadence.^67^ Incorporating WBV into static exercises also increased muscle activation across various leg muscles, regardless of motor impairment levels.^66^

These findings collectively highlight WBV as a promising intervention for improving muscle strength, reducing spasticity, and enhancing functional outcomes in poststroke rehabilitation.

### Effect of repeated muscle vibration therapy and WBV on SCI and CPal: pain, spasticity, and motor recovery

Research supports the effectiveness of WBV and repeated muscle vibration (rMV) for managing pain, reducing spasticity, and enhancing motor recovery in individuals with SCI and Cpal. These therapies offer distinct benefits across clinical populations. In this RCT involving 16 patients with chronic SCI who experienced neuropathic pain, the participants underwent either 8 or 16 WBV sessions. Eight sessions significantly reduced neuropathic pain, particularly in those with severe initial pain levels, as measured by the Neuropathic Pain Symptom Inventory. However, the 16 sessions yielded mixed results, with some participants reporting worse symptoms. Reflex excitability changes associated with initial pain levels underscore the importance of tailoring intervention plans.^62^ Another RCT examined the effects of WBV on ankle spasticity, balance, and gait in 28 patients with incomplete cervical SCI. The WBV group demonstrated notable reductions in ankle plantar flexor spasticity, decreased postural sway, and improved walking speed and balance, outperforming the control group.^63^ For children with CPal, rMV can improve orofacial motor control and reduce drooling. In a study involving 22 children who completed a 3-day rMV protocol, assessments over 3 months revealed consistent and significant reductions in drooling. The findings highlight rMV as a noninvasive therapy to enhance proprioceptive input and motor coordination.^75^

These results emphasize the potential of WBV and rMV for addressing the critical challenges in SCI and CPal rehabilitation, including pain management, spasticity reduction, and functional improvement. Further research should refine treatment protocols and evaluate long-term effects to optimize therapeutic approaches.

### Exploring the effects of FMV and WBV on gait and stability in neurologic disorders

**Fig 4:**
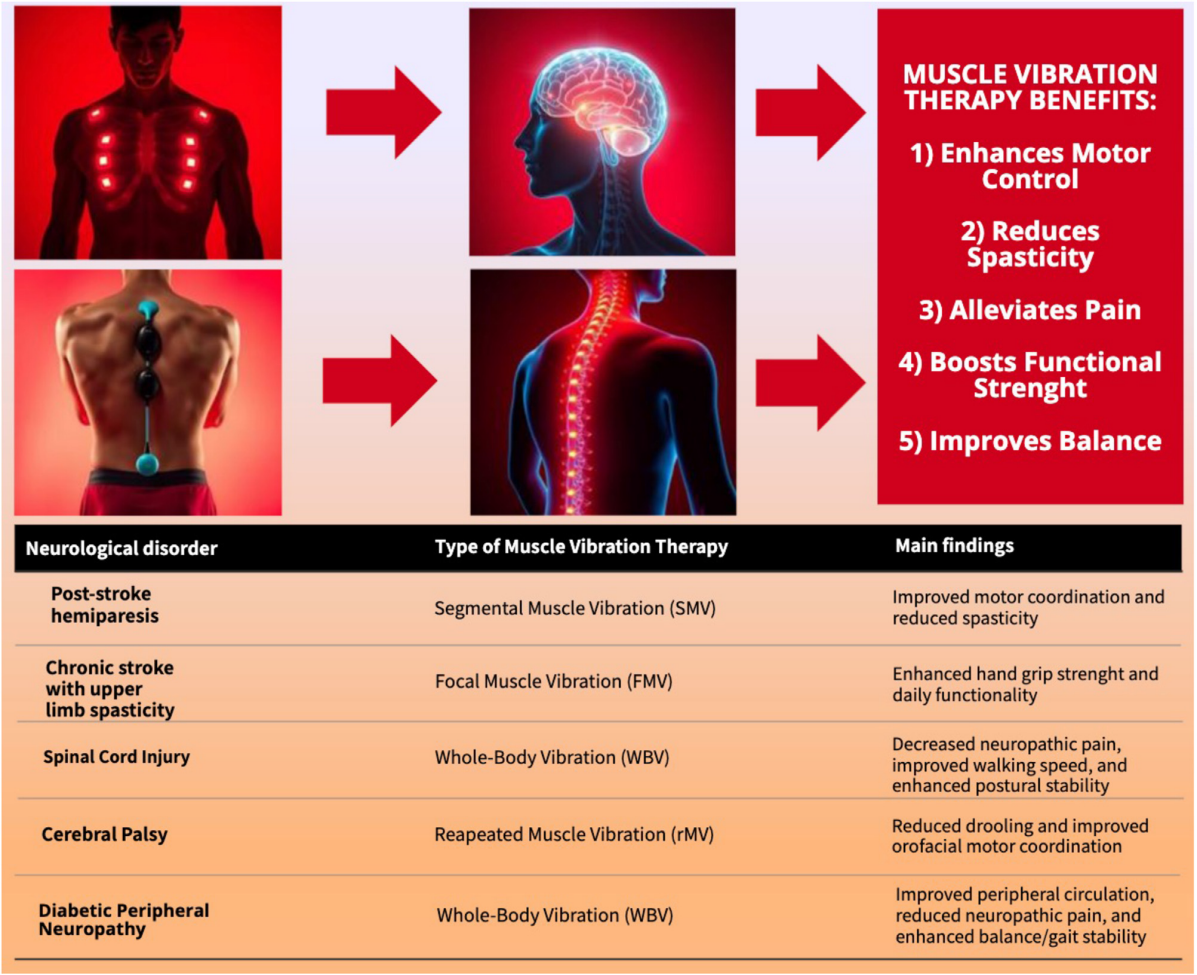
Key findings of the studies on muscle vibration therapy. This figure summarizes the benefits of muscle vibration therapy across various neurologic disorders. The table highlights the type of therapy applied (eg, SMV, WBV) and the main findings for each condition. Benefits include enhanced motor control, reduced spasticity, pain alleviation, improved functional strength, and better balance. Abbreviations: FMV, focal muscle vibration; rMV, repeated muscle vibration; SMV, segmental muscle vibration; WBV, whole-body vibration.

Studies exploring the application of FMV and WBV on gait and stability in neurologic disorders have shown promising outcomes. These approaches have been tested in patients with DPN, MS, and PD, highlighting their potential to improve mobility and reduce fall risks. In a RCT involving 23 individuals with DPN, FMV applied to specific muscles significantly improved gait parameters, including speed, cadence, and peak knee flexor moment during the gait cycle. Enhanced stability during walking was also observed, underscoring the efficacy of FMV in managing mobility impairments in this population.^22^ A study assessing the effects of neck muscle vibration (NMV) on balance and motor skills found notable improvements in weight distribution and functional balance, with no significant differences based on the lesion side.^65^ Yang et al^69^ investigated the effects of WBV on fall risk and bone mineral density in 25 individuals with MS. After 2 months of WBV training, participants demonstrated improvements in balance, functional mobility, muscle strength, and bone density, with moderate to large effect sizes (0.571-1.007). These findings emphasize the potential of WBV to enhance neuromuscular and skeletal health in patients with MS.^69^ Research involving patients undergoing PD focused on cardiovascular responses during static exercises combined with WBV. In an uncontrolled experimental study, 24 older adults with PD performed deep squats on a vibrating platform, significantly increasing systolic blood pressure (10-50 mmHg). This reaction highlights the importance of monitoring cardiovascular stress when implementing WBV in this population.^70^ Leplaideur et al^74^ examined the effects of NMV on posture in patients with hemispheric lesions. Among the 31 participants, NMV caused a significant lateral postural shift toward the paralyzed side, particularly in patients with sensory impairments or visual illusions. However, no changes in anteroposterior placement were observed, highlighting NMV’s localized sensory effect.^74^ A graphical abstract of the key findings of the results section is presented in figure 4.

These studies demonstrated that FMV and WBV positively affect gait mechanics, stability, and neuromuscular function in neurologic disorders. Future research should refine these approaches to ensure safety and maximize therapeutic benefits.

## Discussion

This systematic review explored MV therapies for neurologic conditions, including SMV/LMV and WBV. While WBV targets the entire body, SMV/LMV focuses on specific muscles; both aim to enhance motor and functional recovery. Findings suggest MV interventions can improve motor function, reduce spasticity, and relieve pain, showing moderate promise in neurorehabilitation. MV often enhances outcomes when combined with other therapies, demonstrating versatility in clinical settings.^22^^,^^53^, ^54^, ^55^, ^56^, ^57^, ^58^, ^59^, ^60^, ^61^, ^62^, ^63^, ^64^, ^65^, ^66^, ^67^, ^68^, ^69^, ^70^, ^71^, ^72^, ^73^, ^74^, ^75^, ^76^

### Enhancing precision in neurorehabilitation: SMV and LMV

SMV and LMV can offer specific advantages in the rehabilitation of neurologic conditions, particularly after a stroke.^78^ Research has demonstrated the ability of hemiparetic limbs to improve motor function by decreasing spasticity and enhancing coordination.^79^, ^80^, ^81^, ^82^ For example, SMV has been shown to improve upper limb motor control by reducing cocontraction and optimizing muscle activation patterns, particularly when combined with physical therapy.^54^ RCTs have highlighted some improvements in functional outcomes, such as grip strength and range of motion, when SMV is paired with robotic-assisted therapy or modular rebalancing interventions.^83^ Similarly, LMV effectively reduces spasticity, enhances grip strength, alleviates pain, and improves daily functionality in stroke patients.^80^ These findings demonstrate that segmental and local vibrations can be precise and effective methods for targeted rehabilitation, meeting individual patient needs. In addition to providing immediate functional benefits, the SMV and LMV are vital in promoting neuroplasticity, which is essential for long-term recovery. Vibrations can enhance proprioception, improve sensory input, and facilitate cortical reorganization.^84^ Neuroimaging techniques, including functional magnetic resonance imaging, have revealed that these interventions activate the somatosensory and motor cortices, strengthening synaptic connections and enabling functional recovery.^81^^,^^85^, ^86^, ^87^ Moreover, SMV- and LMV-targeted natures foster localized neuroplasticity by stimulating specific muscle groups. These interventions help reduce maladaptive spasticity and promote adaptive motor recovery by balancing excitatory and inhibitory signals within the central nervous system.^12^^,^^36^ The precision and efficacy of SMV and LMV make them integral to achieving immediate clinical improvements and supporting the biologic processes necessary for sustained rehabilitation success.

### Global impacts: WBV and rMV

WBV and rMV provide systemic and localized benefits, addressing spasticity, balance, and pain while promoting neuromuscular coordination. WBV activates the neuromuscular system across the body, thereby improving lower limb function, postural control, muscle strength, and blood flow in poststroke populations.^88^^,^^89^ Combining WBV with task-specific training amplifies these effects, enhancing functional mobility and reducing spastic hypertonia.^90^^,^^91^ rMV offers targeted benefits for specific conditions, such as Cpal and SCI. It has shown promise in improving orofacial coordination, controlling drooling in Cpal, modulating reflex sensitivity, and reducing neuropathic pain in patients with SCI.^75^ WBV and rMV facilitate neuroplasticity, driving adaptive neural changes crucial for functional improvement.^39^ WBV enhances interhemispheric communication by coordinating sensorimotor circuits on both sides of the body.^92^ This synchronization is essential in poststroke rehabilitation, where cohesive neural responses are needed to restore balance and motor control. rMV recalibrates motor patterns by modulating spinal and supraspinal pathways, reducing excessive reflex responses, and improving motor fluidity.^93^ Together, WBV and rMV extend beyond localized effects, driving broader neuroadaptive changes and advancing their roles in neurorehabilitation environments.^94^

### Emerging areas in physical therapy: the effect of robotics, ai, and mv on motor function

The combination of MV with robotics and artificial intelligence (AI) represents a developing field in neurorehabilitation; however, the findings from our review indicate a more nuanced reality. Claims of enhanced neuromuscular activation and personalized therapy through AI-driven vibration adjustments^95^, ^96^, ^97^, ^98^ are theoretically compelling, but the practical implementation and validation within clinical trials remain limited. Notably, our review found that studies predominantly focused on the individual effects of SMV, FMV, and WBV across various neurologic conditions, with a relative scarcity of research directly assessing the combined effect of robotics and AI. For instance, although robotic-assisted rehabilitation with vibrotactile feedback showed promise in improving motor control,^56^ the studies did not extensively explore the dynamic, AI-driven adaptation of vibration parameters in real-time, as suggested in broader literature. This gap highlights a critical disconnect between theoretical advancement and empirical validation. Furthermore, the variability in study designs, sample sizes, and outcome measures across our included studies underscores the challenges in drawing definitive conclusions about the synergistic effects of robotics and vibration. Moreover, the observed improvements in spasticity, motor function, and gait metrics, while significant in many cases, often relied on traditional statistical analyses like nonparametric tests and *t* tests, with moderate effect sizes.^22^^,^^52^, ^53^, ^54^, ^55^, ^56^, ^57^, ^58^, ^59^, ^60^, ^61^, ^62^, ^63^, ^64^, ^65^, ^66^, ^67^, ^68^, ^69^, ^70^, ^71^, ^72^, ^73^, ^74^, ^75^, ^76^ Although these findings support the therapeutic potential of MVT, they also highlight the need for more sophisticated analytical approaches that can capture the complex interactions between robotic interventions and personalized vibration protocols. Compared with the broader literature, which highlights the potential of AI to tailor rehabilitation interventions through real-time data analysis and adaptive algorithms,^99^, ^100^, ^101^ our review reveals that the clinical application of these technologies in conjunction with MV is still in its nascent stages. While studies have suggested that WBV can improve stability and coordination,^22^^,^^69^ the integration of AI to dynamically adjust these protocols based on individual patient responses remains largely unexplored within the studies we reviewed. Future research should focus on developing standardized protocols, incorporating advanced AI algorithms for real-time adjustments, and employing more sophisticated analytical techniques to fully elucidate the synergistic effects of these technologies in neurorehabilitation. Table 1 summarizes the advantages of various MV types in neurologic conditions.

**Table 1:** Descriptions and advantages of muscle vibration therapies.

Type of Vibrations	Definition	Mechanism of Action	Intervention Duration and No. of Sessions	Neurologic Disorders for Applications (Based on Findings)	Benefits and Practical Application of the Technique	Integration with Robotics and Artificial Intelligence
Segmental muscle vibration (SMV)	A form of vibration therapy used on a specific muscle area or group, commonly employed to improve proprioceptive feedback and motor control.	Activates muscle spindles to improve motor coordination, decrease spasticity, and enhance muscle tone.	Consists of a therapy duration of 8-12 wk, including sessions that occur 2-3 times/wk.	Poststroke hemiparesis, spasticity in upper limbs.	Enhancements in motor coordination, decreased stiffness, and boosted muscle strength. Improves everyday performance and enhances motor coordination.	Enhances targeted motor training when integrated with AI-driven robotic systems, improving movement precision and reducing therapy duration through optimized neuroplasticity.
Local muscle vibration (LMV)	Applying vibration to a specific muscle area to address particular motor issues or spasticity.	Activates neuromuscular pathways, reduces hypertonicity, and improves muscle activation and joint control.	4-6 wk of treatment with 2-3 sessions/wk; each session lasting 10-20 min.	Chronic stroke, upper limb hemiparesis, and spasticity.	Enhanced hand grip strength, reduction in spasticity, decreased pain, and improved motor function (eg, Fugl-Meyer and Quick DASH scores).	Robotic integration allows precise targeting of small muscle groups with AI-modulated vibration frequencies, resulting in improved joint control and proprioception.
Repeated muscle vibration (rMV)	Application of repetitive vibrations to a specific muscle or muscle group for a prolonged period to induce adaptive neuromotor responses.	Modifies proprioceptive input to improve muscle control and sensory-motor integration.	Usually involves short bursts of vibration repeated over several sessions (eg, daily or weekly).	Cerebral palsy, orofacial coordination, and drooling control.	Notable improvement in motor coordination, reduced drooling, and enhanced orofacial control in children with cerebral palsy.	Robotic systems use rMV to enhance feedback-based therapy, particularly in children with cerebral palsy, for fine-tuning motor coordination in real-time.
Whole-body vibration (WBV)	A therapeutic approach where vibrations are applied to the whole-body, often using a vibrating platform, to improve overall neuromuscular performance.	Enhances blood flow, decreases spasticity, and activates large muscle groups for improved motor performance and balance.	Interventions typically last 4-8 wk, with 3-5 sessions/wk.	Poststroke spasticity, spinal cord injuries, and lower limb dysfunction.	Improved muscle strength, balance, and motor control. Significant reduction in spasticity and enhancement in functional mobility and walking speed.	AI-integrated WBV adjusts vibration based on EMG feedback, optimizing neuromuscular performance. Robotic support ensures stability during sessions, improving safety and effectiveness.
Focal muscle vibration (FMV)	A precise vibration technique targeting specific muscle groups to improve localized motor functions or reduce spasticity.	Provides targeted proprioceptive input to enhance motor coordination and decrease muscle resistance during movement.	4-6 wk of therapy, typically involving daily or alternate-day sessions of short duration.	Chronic stroke, hemiparesis, and upper limb dysfunction.	Reduction in spasticity, improved motor function (eg, Wolf Motor Function Test), enhanced muscle activation, and decreased cocontraction in muscle pairs.	Robotic exoskeletons integrate FMV to focus on specific neural pathways, using AI algorithms to adapt vibration parameters in real-time for optimal rehabilitation outcomes.

Abbreviations: AI, artificial Intelligence; EMG, electromyography; FMV, focal muscle vibration; LMV, local muscle vibration; QuickDASH, shortened Disabilities of the Arm, Shoulder and Hand Questionnaire; rMV, repeated muscle vibration; SMV, segmental muscle vibration; WBV, whole-body vibration.

### Study limitations

This systematic review addresses the essential strengths and limitations that influence the overall effect of neurorehabilitation with MVT. The inclusion of 787 participants from 26 studies provides a solid foundation for analyzing the effects of MVT on various neurologic conditions. However, the variability in sample sizes, ranging from 15-80 participants, and differences in study methodologies may affect the consistency and comparability of outcomes. This review’s main strength lies in its thorough search strategy, which involves searching through databases such as PubMed, Web of Science, and Embase to ensure a comprehensive selection of relevant literature. This comprehensive method improves the accuracy of the results, leading to a more robust comprehension of MVT in different neurologic conditions. Using the PRISMA guidelines for systematic reviews enhances methodological rigor, promoting transparency and reproducibility. Moreover, using the PICO framework for creating search terms enables targeted investigation of pertinent literature, thereby aiding in the discovery of crucial studies highlighting the effectiveness of MVT in neurorehabilitation. Moreover, using tools like RoB 2 and ROBINS-I to evaluate bias risk offers valuable insight into the methodological rigor of studies on MVT, enhancing the assessment of study quality.

However, despite these strengths, there are clear limitations. A significant drawback of the included studies is methodological issues, particularly regarding the randomization procedures and the choice of presented findings. The discovery of significant biases in certain studies calls into question the trustworthiness of their conclusions, indicating that discretion should be exercised in analyzing the outcomes. Moreover, nonrandomized studies have moderate to severe biases, which suggests that although they offer valuable insights, their methodological limitations could affect the broad applicability of the findings. Although an extensive search strategy was used on significant databases, the study only included research over a period of 2014-2024, possibly missing essential studies before that period. Another restriction is found in the diversity of research plans and sample sizes, which can affect the comparability of outcomes. The wide array of neurologic disorders considered could also hinder the integration of results because the effect of MVT may vary significantly among different conditions. Another constraint is the omission of databases such as the Cochrane Library and Scopus from the article search, resulting in the retrieval of only 26 articles for analysis. Using bibliographies from different systematic reviews to find studies is another constraint, because it could affect repeatability, which is crucial, and may also lead to bias. Future studies need to aim for more consistency in methodology and clarity in reporting to expand on the results of this review and improve the trustworthiness of conclusions about the effectiveness of MV in neurorehabilitation.

## Conclusions

In conclusion, this systematic review emphasized the potential benefits of MVT for neurorehabilitation in different neurologic conditions. Incorporating MVT into rehabilitation programs has demonstrated positive effects on motor control, spasticity reduction, and functional outcomes for patients. Nevertheless, the review also recognized the necessity for stricter methodologies in upcoming studies to reduce the recognized biases and improve the dependability of results. Future studies should prioritize conducting more extensive RCTs with standardized protocols to evaluate the effectiveness of various vibration techniques in different patient groups. Moreover, investigating how MVT works so that it can eventually be combined with other forms of rehabilitation, such as robotics and AI, may result in unique treatment methods.

## Suppliers

a. Excel; Microsoft.
b. RoB 2; Cochrane.

## Disclosure

The investigators have no financial or nonfinancial disclosures to make in relation to this project.

## Appendix Supplementary materials

Image, application 1
