# Supplementary material for: Exploring the Impact of Muscle Vibration Therapy in Neurologic Rehabilitation: A Systematic Review
Source: Arch Rehabil Res Clin Transl. 2025 Jun 6;7(3):100478. doi: 10.1016/j.arrct.2025.100478 (PMC12447217; doi:10.1016/j.arrct.2025.100478)
Supplement: Supplementary file 1 [file mmc1.docx]

**Table 1.** Summary of studies included in the research.

| **Author** | **Aim** | **Study Design/Intervention** | **Treatment Period** | **Sample Size** | **Sample Characteristics/Clinical Outcomes** | **Outcomes Measures** | **Main Findings** | **Effect Size/Certainty of Evidence** | **Statistical Analysis/Magnitude of Effect** |
| --- | --- | --- | --- | --- | --- | --- | --- | --- | --- |
| Annino et al. 2019 [44] | To study how SMV and SPT work together on functional outcomes in post-ischemic stroke patients. | Randomized Controlled Trial. | 8 weeks. | 37 participants were initially registered, with 34 successfully finishing the research. | *Age*: not Specificated  *Gender*: 7 females; 30 males.  *Clinical outcomes:* Improvement in function and muscle stiffness in the paralyzed arm. | BI for ADL; MAS, MMT and goniometry for ROM. | Both sets exhibited notable enhancements in BI, ROM, and muscle strength. Yet, only the group undergoing experimentation showed notable enhancement in muscle tone (decreased spasticity) in the hemiplegic elbow joint. | Effect Size: Moderate to substantial in various aspects: BI (SPT-SMV: 0.62, SPT: 0.61), ROM (SPT-SMV: 0.46, SPT: 0.43), Elbow Tone (SPT-SMV: 0.45, SPT: 0.30).  Certainty of Evidence: Moderate was backed by statistical significance in multiple results, yet no notable variances were observed between the groups by the end of the treatment. | Nonparametric tests (Mann–Whitney U, Wilcoxon signed-rank). Effect sizes were calculated using the formula 𝑟=𝑍/𝑁r=Z/ N. |
| Celletti et al. 2017 [45] | Assessing the impact of FMV when paired with RMP or CP on motor function, spasticity, and pain among long-term stroke survivors. | Randomized Controlled Trial. | 6 weeks, with FMV applications over 3 consecutive days. | 18 patients. | *Age*: not Specificated  *Gender*: not Specificated  *Clinical outcomes:* Enhancement of motor function, reduction in spasticity, and improvement in pain levels in the impacted upper limb. | WMFT, MAS, VAS, MI. | Both FMV+RMP and FMV+CP groups exhibited notable enhancements in WMFT, MI, and VAS results after treatment, whereas only Group 3 (CP) did not show any improvement in WMFT scores. Scores in the MAS significantly increased for all three groups. | Effect Size: not Specificated.   Certainty of Evidence: Moderate, considering the limited sample size and experimental design of the pilot study. | The Wilcoxon signed-rank test was employed to assess changes in outcome measures. Both treatment groups (FMV+RMP, FMV+CP) showed important changes with P<0.05 for all tests. |
| Paoloni et al. 2014 [46] | To assess how low-amplitude SMV impacts muscle activation and movement control in chronic stroke patients with hemiparesis. | Randomized Controlled Trial. | Participants receive treatment five times a week for a duration of two weeks. | 22 individuals with hemiparetic stroke (12 in the experimental group, 10 in the control group) were included, along with 10 healthy subjects of similar age for normative data collection. | *Age*: 40 to 85, while healthy subjects have an average age of 60.  *Gender*: both males and females  *Clinical outcomes:* Enhancement in motor function, especially in muscle activation time and CCI during reaching actions. | Brunnstrom’s Stage, FMAS, MAS, EMG. | The experimental group showed notable enhancements in muscle initiation times for specific muscles, decrease in CCI for certain pairs of muscles, improved control of muscle activation, and a reduction in %MVC for the BB muscle after treatment, when compared to the control group. There were no negative incidents seen in both groups. | Effect Size: There are notable variations in the timing of muscle activation, as seen in posterior deltoid (total ranks = 6.00; p = 0.03) and biceps brachii (t = 2.75; p = 0.02).   Certainty of Evidence: High, backed by strong statistical analysis and intention-to-treat principles. | D'Agostino-Pearson test for assessing normality, t-tests or Mann-Whitney tests, Bonferroni correction to reduce Type I error. Important discrepancies were observed between pre-treatment and post-treatment in the experimental group (e.g., p < 0.05). |
| Costantino et al. 2017 [47] | To assess how well local mechano-acoustic vibration therapy helps decrease upper-limb spasticity and enhance muscle strength, mobility, and daily functioning in individuals with chronic stroke. | Randomized Controlled Trial. | 12 sessions over 4 weeks, with therapy applied three times per week. | 32 participants. | *Age*: Mean age 61.59 ± 15.50 years.  *Gender*: 21 males, 11 females.  *Clinical outcomes:* Chronic upper-limb spasticity, hemiplegia, positive pathological reflex in the affected limb. | Hand Grip Strength Test, MAS, QuickDASH Score, FIM, FMAS, JTT, VNRS. | Group A, the vibration therapy group, demonstrated significant improvements in hand grip strength, decreased spasticity in the shoulder, elbow, and wrist, improved daily functionality as measured by QuickDASH score, increased self-sufficiency according to FIM, and enhanced upper-extremity sensorimotor function using FMAS, in comparison to Group B, the sham group. | Effect Size: Notable differences were noted in Hand Grip Strength (P=0.001-0.005) and spasticity (P<0.05).   Certainty of Evidence: Moderate certainty based on strong statistical analysis, yet constrained by a small sample size. | Comparisons at the starting point: Single-factor ANOVA. Effects of treatment assessed using two-way repeated-measures ANOVA. P-value less than 0.05 and 95% confidence interval are utilized to establish significance. |
| Calabrò et al. 2017 [48] | To assess how effective combining MV with robotic arm training using the Armeo-Power device is in decreasing upper limb spasticity and improving functional recovery in post-stroke individuals. | Randomized Controlled Trial. | Sessions occur every day, five days a week, for eight weeks in a row (totaling 40 sessions). | 20 patients. | *Age*: 50 to 80 years.  *Gender*: not Specificated.  *Clinical outcomes:* Enhancing use of upper extremities and decreasing muscle stiffness in stroke survivors. | MAS, SICI, HMR, FMA-UE, FIM, HRS-D, HRS-A, EMG. | The main goal was to lower MAS scores, showing improvements in SICI and HMR measures, indicating improved reduction of spasticity. Secondary results demonstrated enhancements in FIM and upper limb motor performance (FMA-UE). EMG and kinematic assessments verified improvements in muscle activation and coordination of movement. | Effect Size: Enhancements in MAS, SICI, and HMR indicate significant changes with clinical relevance.   Certainty of Evidence: Moderate, as a result of the limited number of participants and the preliminary nature of the study. | The minimal detectable change is a 1-point decrease in MAS, which is considered clinically significant. A decrease of 15% in SICI and HMR is seen as significant alterations. Analysis carried out using suitable statistical methods for matched data. |
| Silva et al. 2014 [49] | To study the impact of vibration training on muscle activation and functional mobility in post-stroke patients with hemiparesis. | Randomized Controlled Trial. | January to September 2011. | 43 participants. | *Age*: 20 years or older.  *Gender*: both male and female participants.  *Clinical outcomes:* Progress in motor skills and physical movement in individuals recovering from a stroke and experiencing weakness on one side of their body. | FMA, MMSE, EMG, SCT, 6MWT, TUG. | The intervention group showed lower functional mobility and muscle activation, with shorter mean distances in the 6MWT and longer times in the TUG compared to the control group. There were no notable variations seen amongst the groups in regards to enhancements in motor function or the relationship between time and group on the primary outcome measures. | Effect Size: Not Specificated.   Certainty of Evidence: Moderate; backed by strong methodology but constrained by inconsistency and absence of notable impacts. | Independent t-test for baseline comparisons. Paired t-test for intragroup differences. ANCOVA for time and group interactions. Significance Level: 𝑝<0.05 |
| Yang et al. 2021 [50] | To examine how the muscle strength and bone turnover in older adults with stroke are impacted by WBV at varying frequencies. | Randomized Controlled Trial. | Over a period of 8 weeks, participants will engage in 24 training sessions, with each session occurring 3 days per week. | There were 80 participants divided evenly into two intervention groups, with 40 individuals in each group. | *Age*: 50 years or older.  *Gender*: both male and female participants.  *Clinical outcomes:* Strength of muscles and bone remodeling in individuals who have experienced a stroke. | FMA, MAS, Isokinetic knee muscle strength test, NTx, 6MWT. | The research revealed that muscle strength and bone turnover were affected differently by various frequencies of WBV. | Effect Size: Marked progress in muscle strength and bone turnover markers observed in both frequency groups.  Certainty of Evidence: The trial was moderate, following CONSORT guidelines and involving stratified randomization, with possible limitations including convenience sampling and a small sample size. | AN alternative to ANOVA could be mixed-effects models when comparing groups. |
| Tankisheva et al. 2014 [51] | To assess the impact of a training regimen using a vertical vibration platform on individuals with chronic stroke in a rehab environment. | Randomized Controlled Trial. | 6 weeks, with a subsequent follow-up after 6 weeks. | 15 participants. | *Age*: Ages ranging from 40 to 75 years.  *Gender*: not Specificated.  *Clinical outcomes:* patients who have suffered from a long-lasting stroke and can stand on their own for a minimum of 20 minutes. | MAS, Isokinetic dynamometer tests, SOT, BI, FAC. | Adherence to the vibration treatment was outstanding (100%), without any documented negative outcomes. Participants were able to withstand both vibration frequencies and noted a moderate level of effort, showing that the intervention is feasible and safe. | Effect Size: Isometric and isokinetic knee extension strength showed medium to small differences; the effect size was 1.52 for isometric extension and 1.77 for isokinetic extension.  Certainty of Evidence: Low to moderate because of the limited number of participants and the use of a pilot study design. | Significant differences were found between groups for strength of the paretic leg in both isometric and isokinetic knee extension exercises (P=.022 and P=.005, respectively). |
| Lee et al. 2016 [52] | To assess how WBV training in conjunction with TRT impacts upper extremity functional performance and motor control in poststroke hemiplegia patients. | Randomized Controlled Trial. | 4 weeks. | 45 participants. | *Age*: not Specificated.  *Gender*: 21 females and 24 males.  *Clinical outcomes:* Assessment of poststroke hemiplegia by measuring functional performance and motor control in the upper limbs. | FMS, WMFT, Jamar hydraulic hand dynamometer, MAS. | Notable enhancements in FMS scores were noted in both the WBV group and the WBV plus TRT group in comparison to the control group after the intervention. The group that received WBV plus TRT demonstrated notable enhancements in WMFT scores in comparison to both the control group and the WBV group. The WBV plus TRT group showed notable decreases in spasticity (MAS scores) compared to the control group and WBV groups. | Effect Size: not Specificated.   Certainty of Evidence: Moderate confidence, supported by a randomized design and dependable outcome measures, though constrained by a small sample size and absence of blinding in certain measures. | The analysis involved the use of one-way ANOVA, repeated-measures ANOVA, and Bonferroni post-hoc tests. The extent of impact can be deduced by observing notable variations in upper extremity function and grip strength between the intervention groups and the control group following a 4-week period. |
| Huang et al. 2020 [53] | To examine the immediate impacts of WBV on H-reflex, blood flow in leg muscles, and passive stiffness in the leg muscles of individuals with a history of stroke. | Randomized Controlled Trial. | Participants underwent two separate laboratory assessment sessions, with a minimum of 24 hours and a maximum of 72 hours between each session. | 36 participants. | *Age*: 18 years old or above.  *Gender*: both male and female.  *Clinical outcomes:* Participants had been diagnosed with hemispheric stroke for over 6 months and were in a stable medical condition. | Measurements of the H-reflex were conducted by stimulating the tibial nerve through percutaneous methods. Using ultrasound to evaluate muscle stiffness and blood flow in the MG muscle. | The research showed that WBV had a notable impact on the H-reflex response, blood flow, and passive stiffness of leg muscles in chronic stroke patients. | Effect Size: The anticipated impact of WBV on muscle rigidity was moderate (f=0.25), while H-reflex demonstrated moderate to large effects (Cohen’s d of 0.70–0.90 for reducing H/M ratio).   Certainty of Evidence: Moderate, considering the study design and the accuracy of the measurements. | ANOVA analysis demonstrated significant alterations in H-reflex and muscle parameters following WBV with an alpha level of 1% and 80% power. |
| Wong et al. 2022 [54] | To evaluate how the severity of pain and reflex excitability in individuals with chronic neuropathic pain from SCI are affected by different doses of WBV. | Randomized Controlled Trial. | The research employed a design including wash-in control, intervention, and wash-out, with measurement timepoints at T1-T4. | 16 participants. | *Age*: Not Specificated.  *Gender*: Not Specificated.  *Clinical outcomes:* Individuals exhibited neuropathic pain symptoms at a location below the site of their injury. | NPSI, NRS, MPI-PS, Pendulum test, Modulation of the soleus H-reflex. | The group that did 8 bouts of WBV showed a notable drop in NRS scores from T1 to T2, and both NPSI and MPI-PS scores decreased with WBV from T2 to T3. The results indicated that 8-sessions of WBV therapy may offer greater benefits for those with moderate to high neuropathic pain, while 16-sessions could potentially worsen pain in individuals with moderate to severe symptoms. | Effect Size: The Cohen's d value for NPSI was 0.50, suggesting a moderate effect on pain reduction in the group that underwent 8 bouts of WBV. The effect size for pain severity reduction with MPI-PS was 0.69, indicating a medium to large effect.  Certainty of Evidence: Moderate. The study's exploratory nature, along with its small sample size, could restrict the strength of the results. | Cohen's d was reported for changes across timepoints using paired and independent t-tests. |
| In et al. 2018 [55] | To assess how WBV training impacts spasticity, postural sway, and walking ability in patients with iSCI. | Randomized Controlled Trial. | 8 weeks. | Out of the 38 patients who were initially chosen, 32 met the criteria and 28 finished the intervention. | *Age*: WBV group: 46.1 ± 9.8 years; Control group: 49.9 ± 9.3 years.  *Gender*: WBV group: 9 males, 5 females; Control group: 10 males, 4 females.  *Clinical outcomes:* Individuals with grade D AIS who have iSCI in cervical levels 6 or 7. | Spasticity was evaluated with a hand-held dynamometer; postural sway was analyzed using a force plate device. TUG assessment evaluates walking capability; 10MWT measures walking velocity. | The WBV and control groups both demonstrated notable enhancements in spasticity, postural sway, TUG, and 10MWT following the intervention. There were notable variations in plantar flexor spasticity, postural sway length, TUG, and 10MWT between the WBV and control groups, showcasing strong correlations among these parameters. | Effect Size: not Specificated.   Certainty of Evidence: Moderated with the backing of double-blind methodology and statistical significance. | ANOVA with factorial design, t-tests for paired samples, and correlation analysis using Pearson's r coefficients. Notable interaction effects were seen between the type of intervention and the time for every outcome measure. |
| Julliand et al. 2024 [56] | To evaluate how effective LMV are in decreasing spasticity in stroke patients with upper-limb motor impairments. | Randomized Controlled Trial. | During a span of 6 weeks, sessions will be held 3 times a week. | 100 participants. | *Age*: not Specificated.  *Gender*: both male and female participants.  *Clinical outcomes:* The primary clinical outcome is the level of spasticity assessed by the MAS. | MAS, FMA-UE, TMS. | The research suggests that the group receiving the intervention will have a lower level of spasticity than the control group (15% vs. 40%), with a more significant impact because the intervention lasted longer than in previous studies. | Effect Size: Although not explicitly stated, it is believed that there was a significant improvement in MAS and FMA-UE scores, as suggested by previous research.  Certainty of Evidence: Moderate, backed by previous meta-analyses and adherence to protocol in both centers. | For comparing groups, use either χ² or Fisher's exact test with categorical variables. Quantitative evaluations: Student's t-test or Mann-Whitney U test. ANOVA for within-group time effects using repeated measures. |
| Rippetoe et al. 2020 [57] | To assess the impact of a four-week remote intervention utilizing a adapted wearable gadget that administers FMV on walking characteristics in people with diabetes and diabetic peripheral neuropathy. | Randomized Controlled Trial. | 4 weeks. | 23 individuals. | *Age*: adults aged 18 years and older.  *Gender*: 14 females and 9 males.  *Clinical outcomes:* Having diabetic peripheral neuropathy can be detected by not being able to feel the 5.07 (10 g) monofilament at ten different sites. | Measured gait parameters included speed, length and width of stride, time of stride, stance time for left and right legs, swing time for left and right legs, cadence, duration of double support, peak knee flexion for left and right legs. | Six spatiotemporal gait parameters showed notable enhancements, with a 0.09 second increase in gait speed recorded. Stride time was reduced by 0.08 seconds. Cadence rose by 6.5 steps per minute. The time spent in the left stance was reduced by 0.06 seconds. The duration of the correct posture decreased by 0.08 seconds. Double limb support duration reduced by 0.05 seconds. In addition, there were observed trends towards notable alterations in peak knee flexion and significant rises in peak knee flexor moment. | Effect Size: not Specificated.   Certainty of Evidence: Preliminary conclusions from pilot study may have limited applicability. | Paired t-tests and Wilcoxon signed-rank tests were utilized to compare within the same groups. Comparison of changes between vibration groups was conducted using two-way mixed ANOVA. The significance threshold was established at alpha = 0.05. |
| Jamal et al. 2020 [58] | To assess how NMV impacts postural control and balance in chronic stroke patients with RBD and LBD. | Uncontrolled Experimental Study. | 2 weeks, with sessions held on five days each week. | 39 participants were initially part of the study; nevertheless, only 32 individuals were considered for the final analysis following exclusions. | *Age*: Average age of 60.9 ± 10 years.  *Gender*: 26 men and 6 women.  *Clinical outcomes:* improvements in motricity | WBA, posturography parameters, SSA, LBA, SVV, Motricity Index, MAS, bell cancellation test, TUG, BBS. | WBA exhibited a notable interaction between lesion side and time, showing a decrease in RBD patients specifically on day 15. There was a notable enhancement in TUG and BBS scores on day 21 and day 45. There were no notable differences detected in sensitivity test results or balance ratings among the two groups. | Effect Size: Effect sizes for improvement in WBA in RBD patients are implied to be significant, despite not being directly stated. Additional posturography impacts were substantial with repeated measurements (e.g., SVV deviation P = 0.0005).  Certainty of Evidence: Moderate certainty is present because of the lack of a control group and a small sample size, however, there is strong methodological rigor in the assessment of outcomes. | An investigation of long-term changes was conducted using ANOVA for repeated measures and generalized linear models, followed by Tukey's post-hoc tests to determine significance. Significance is determined by a p-value threshold of 0.05. Significant results: Improvements in WBA were observed in RBD patients (P = 0.05), while reduction in SVV variability was very significant (P = 0.0005). |
| Liao et al. 2015 [59] | To study how the TA and BF muscles on both sides are affected by three various WBV protocols and eight different exercise conditions in chronic stroke patients. | Uncontrolled Experimental Study. | From September 2012 to May 2013. | 36 individuals. | *Age*: Mean age of 57.3 years (SD = 10.7).  *Gender*: 26 men and 10 women.  *Clinical outcomes:* Moderate impairment level of the affected lower limb, as indicated by the CMSA. | CMSA, MAS, EMG. | The research discovered a notable effect of the interaction between exercise intensity and side on the EMG reactions of the TA and BF muscles, suggesting that the response to WBV was affected by the combination of vibration intensity, type of exercise, and limb engagement. Moreover, a higher WBV intensity led to a notable rise in normalized EMG responses in the TA and BF muscles. | Effect Size: Significant effect sizes were observed for WBV intensity alone (f = 0.66–0.93) and for the interaction of intensity with exercise (f = 0.23–0.44).   Certainty of Evidence: High reliability of EMG measurements (ICC = 0.96-1.00) provides strong evidence of internal validity. | Three-part repeated measures ANOVA for limb, WBV intensity, and exercise conditions. Analysis of trends in the effects of WBV intensity and correlations with Spearman's coefficients for EMG responses relating to motor impairment and spasticity scores (CMSA, MAS). Bonferroni corrections were applied in the post hoc comparisons. |
| Miyara et al. 2014 [60] | To assess how WBV impacts lower-limb spasticity and motor function in individuals who have had a stroke. | Uncontrolled Experimental Study. | From June 1, 2011, until September 30, 2012. | 25 patients. | *Age*: Mean age was 52.2 years (range: 20-75 years).  *Gender*: 20 males and 5 females.  *Clinical outcomes:* Patients with hemiplegia experienced lower-limb spasticity, along with different degrees of motor improvement. | MAS, A-ROM and P-ROM for ankle dorsiflexion, 10-MWT. | After the intervention, there was a notable decline in MAS scores for the hip adductor muscles, hamstring muscles, and gastrocnemius muscles. An increase in ankle dorsiflexion range of motion was observed following WBV. No subjects experienced any discomfort during or after the intervention. | Effect Size: Spasticity (MAS): Significant impact (e.g., initial 2.1 ± 0.8, final 1.6 ± 0.7, P = 0.002). A-ROM increased significantly from 5.0 ± 3.9 to 9.6 ± 4.1 (P = 0.001). Speed of walking: Before: 34.4 ± 17.2, after: 36.6 ± 18.2, P = 0.002.  Certainty of Evidence: Low. Initial data from a limited number of participants. | Comparisons were conducted using Wilcoxon signed-rank tests. Notable advancements were seen in all aspects (e.g., spasticity, range of motion, and gait outcomes) with statistical significance (P ≤ 0.05). |
| Liao et al. 2014 [61] | To assess how WBV affects the VL and GS muscles in individuals with chronic stroke while performing various exercises. | Uncontrolled Experimental Study. | Participants completed workouts in three different WBV settings within one session. | 45 patients. | *Age*: not Specificated.   *Gender*: 34 men and 11 women.  *Clinical outcomes:* A long-lasting stroke affecting one side of the brain with weakness in the lower limb (hemiparesis occurring at least 6 months ago). | Bilateral muscle activity of the VL and GS was recorded using surface EMG, expressed as a percentage of EMG amplitude recorded during maximal voluntary contraction. | Being exposed to low- and high-intensity WBV caused a notable increase in the EMG amplitude of VL and GS on both the affected and unaffected sides in different exercise situations, compared to not using WBV, with a significant impact. There was no notable variance in EMG magnitude between the high- and low-intensity WBV protocols. WBV increased muscle activation in both leg muscles equally during various exercises. | Effect Size: Significant impact found for vibration intensity on muscle activation in the weak leg (partial η² = 0.38).  Certainty of Evidence: Moderate level of confidence supported by strong research methods and important results; constrained by small sample size and one-time study design. | Analysis of variance with repeated measures in two directions using partial eta-squared for effect size measurement. Comparison was done using paired t-tests and Bonferroni correction for further analysis. Spearman's rho measured the relationship between spasticity and EMG activity. |
| Yang et al. 2018 [62] | To examine how an 8-week WBV training plan impacts the enhancement of fall risk factors and bone mineral density in community-dwelling adults with MS. | Uncontrolled Experimental Study. | 8 weeks. | 25 participants. | *Age*: not Specificated.  *Gender*: not Specificated.  *Clinical outcomes:* Participants were diagnosed with MS by a neurologist and had a PDDS score of less than or equal to 6.5. | EquiScale Test for body balance; TUG; isokinetic dynamometry; goniometer; FESI; bone ultrasonometer at the calcaneus for bone mineral density. | After training, there was a notable enhancement in several fall risk factors, such as a rise in EquiScale scores. Shortened duration for TUG test completion. Lower fear of falling scores were observed. Noteworthy enhancements were observed in the strength of the knee extensors and knee flexors on the weaker side. Enhanced flexibility in both pointing and flexing the foot at the ankle joint. Improved bone density observed at both strong and weak sides of the calcaneus. | Effect Size: Cohen's effect sizes ranged from 0.114 for knee extensor strength to 1.007 for balance, showing different levels of improvement, with some factors displaying significant effects.  Certainty of Evidence: The level of significance was high for all individuals who completed the entire 8-week training period, with statistically significant results seen in various tests. | Paired t-tests and Hotelling's T2 test showed meaningful enhancements (p-values ≤ 0.038), with effect sizes for different measures ranging from moderate to substantial (Cohen’s dz 0.114 to 1.007). |
| Niewiadomski et al. 2023 [63] | To examine how WBV paired with static exercises impacts BP reactions in PD patients. | Uncontrolled Experimental Study. | Not specificated the period but the session included six repetitions of static squats for the exercises. | 24 participants. | *Age*: 55–75 years.  *Gender*: 12 male and 12 women.  *Clinical outcomes:* The research concentrated on how BP reacts when doing static squats on a vibrating platform. | SBP, DBP, PP, HR, ECG. | There was a notable increase in BP during the use of the vibrating platform, specifically during deep squats where the highest elevation was recorded. The rise in SBP and DBP peaked during the third round of the exercises. The extent of PP rise varied depending on the exercise type, with deep squats resulting in noticeably higher values than calf exercises. The HR also increased significantly, with the biggest jumps seen during deep squats. | Effect Size: Notable variations in cardiovascular reactions (SBP, DBP, PP, HR) were noted among different types of exercises, particularly in deeper squats and for individuals who are more reactive.  Certainty of Evidence: Moderate to high, determined from strong statistical analyses and notable differences within groups. | Significant impacts of exercise type and repetition on SBP, DBP, PP, and HR were observed in the Two-way ANOVA for repeated measures, with F-values of 24.559, 25.874, and 12.037 respectively, all having p < 0.001. The regression analysis showed significant differences in the cardiovascular reaction among individuals, particularly in SBP across various squat variations. |
| AhmadAbadi et al. 2018 [64] | To assess the impact of active vibration compared to fake vibration on balance, ankle plantar flexor spasticity, and ankle dorsiflexion PROM in individuals who have had a stroke and are dealing with spastic hemiplegia. | Comparative Study. | Two therapy sessions were held one week apart. | 22 participants. | *Age*: Mean age of 55.82 ± 11.87 years.  *Gender*: 8 females and 14 males.  *Clinical outcomes:* Spastic hemiplegia caused by a stroke, lasting at least six months post stroke. | Mini-BESTest, MMAS, ankle PROM. | Active vibration therapy was shown to enhance balance and decrease ankle plantar flexor spasticity in stroke patients, as indicated by improved Mini-BESTest scores and lower MMAS scores post-treatment. The enhancements were significantly higher when using active vibration instead of placebo. | Effect Size: The effect size was 0.85 for Mini-BESTest, 0.14 for placebo vibration, and 0.30 for dorsiflexion PROM after active vibration, as measured by Cohen's d.  Certainty of Evidence: High, as a result of the notable discoveries and proper statistical evaluation. | Repeated measures ANOVA and Bonferroni post-test were utilized to analyze balance and PROM, while Cohen's d was employed to measure effect size, revealing a substantial effect size for balance (0.85) and a moderate effect size for dorsiflexion PROM (0.30). |
| Miyara et al. 2018 [65] | To assess the impact of WBV treatment on spasticity in the lower limbs of individuals who have had a stroke. | Comparative Study. | Each WBV session lasted 5 minutes, with evaluations carried out before, right after, 10 minutes after, and 20 minutes after each treatment. | 16 patients. | *Age*: Mean age of 54.7 ± 13.5 years  *Gender*: 14 men and 2 women.  *Clinical outcomes:* The main clinical outcomes assessed included F-wave parameters. | MAS, A-ROM and P-ROM. | WBV led to a significant decrease in F-wave amplitudes and F/M ratios, suggesting a decrease in spasticity. MAS ratings of hip adductors, hamstrings, and soleus muscles showed a significant decrease following WBV. Advancements in ankle dorsiflexion range of motion were observed, indicating the potential effectiveness of WBV therapy in treating spasticity in stroke patients. | Effect Size: The effect size (r) varied between 0.63 and 0.87, suggesting a moderate to strong effect.  Certainty of Evidence: Moderate to high levels of statistical significance were found across various measures and time points. | Statistical significance was determined through Friedman and post hoc tests, showing significant decreases in F-wave amplitudes, MAS scores, and ROM measurements, indicating meaningful enhancements in spasticity. |
| Seim et al. 2023 [66] | To assess how well the VTS Glove works in managing spastic hypertonia in chronic stroke patients. | Prospective Study. | The duration was 12 weeks, with an 8-week intervention phase followed by a 4-week post-intervention phase. | 21 patients. | *Age*: 18 years or older.  *Gender*: not Specificated.   *Clinical outcomes:* Subjects exhibited hand spasticity following a stroke, as evidenced by a minimum score of 1 on the MAS. | MAS at fingers, MTS angle at fingers and AROM at fingers. | The results indicated that the VTS Glove treatment significantly influenced spastic hypertonia, as shown by variations in the MAS and MTS scores, and AROM in subjects post the 8-week intervention and throughout the follow-up period. | Effect Size: Significant (0.84) for enhancing MAS.  Certainty of Evidence: High, due to significant statistical results (p=0.0014) and a substantial effect size. | Wilcoxon Signed-Rank tests and paired t-tests showed a substantial effect size (0.84) in reducing MAS symptoms. |
| Leipladeur et al. 2016 [67] | To assess how neck muscle vibration impacts postural reactions in patients who have had a stroke on either the right or left side of their brain. | Prospective Study. | April 2011 to April 2013. | 31 patients. | *Age*: Mean age of 61.5 years (SD 10.6).  *Gender*: 25 men and 6 women.  *Clinical outcomes:* Patients had right-hand dominance and were admitted within 6 months following their first hemispheric stroke. | BI, FAC, Bells test. | Following neck muscle vibration, the average mediolateral center of pressure position notably moved towards the hemiplegic side, suggesting enhanced postural stability. The difference in displacement was 20.7 ± 11.5 mm prior to vibration and 18.5 ± 13.5 mm post-vibration, showing a notable average difference of 3.1 mm. Patients who were vulnerable to visual tricks saw more significant enhancements in displacement compared to those who were not (average displacement change: 4.7 mm vs. −0.08 mm). | Effect Size: Positive average change in CoP position recorded as 3.1 mm, with a statistically significant p-value of less than 0.005.   Certainty of Evidence: Moderate, determined by statistical significance and strong analysis. | Wilcoxon test and mixed model analysis will be utilized, along with applying Bonferroni correction for analyzing multiple comparisons. Statistically significant enhancements observed in CoP position (p < 0.005). |
| Russo et al. 2019 [68] | To assess the efficiency of muscle vibration therapy in decreasing drooling in children with CP. | Prospective Study. | The therapy took place for three days in a row, with three 10-minute sessions daily, spanning from February 2016 to April 2018. | 22 Children. | *Age*: 5 to 15 years (mean 9.28 ± 3.62).  *Gender*: 8 males and 14 females.  *Clinical outcomes:* Decrease in how often, how strong, and how severe drooling is. | DIS, DFSS, VAS, DQ. | Noticeable decreases in drooling occurrence, strength, and seriousness were noticed in all evaluations (10 days, 1 month, and 3 months after treatment) compared to the initial state, with enhancements staying consistent post-treatment. | Effect Size: There was a notable decrease in drooling on all scales (p < 0.001 for all results).   Certainty of Evidence: Moderate, given that this is an initial study with a limited number of participants. | The results of the Friedman test showed significance for all measures of outcome. Post-hoc examination revealed notable disparities in scores between initial and post-treatment assessments (T0 versus T1, T2, T3). |
| Takeuchi et al. 2024 [69] | To assess how effective three various treatments (stretching and vibration therapy) are at decreasing spasticity in stroke patients with hemiparesis in their upper limbs. | Retrospective Observational Study. | The treatments were administered daily for three days in a row, spanning from November 2018 to March 2019. | 27 patients. | *Age*: over 20 years old.  *Gender*: not Specificated.   *Clinical outcomes:* Subjects exhibited reduced strength and atypical muscle tension in the wrist or finger flexors on the affected side (MAS score 1-3). | MAS. | The research discovered that tendon vibration treatment was more successful than stretching or muscle belly vibration in decreasing spasticity in the finger flexors. Furthermore, sustained reduction in wrist flexor spasticity was observed following voluntary finger flexion due to muscle belly vibration. In general, tendon vibration had the best success in reducing spasticity in finger flexors compared to the other interventions studied. | Effect Size: Notable variations were noted in MAS scores and response rates among different treatments, such as P<0.05 and P<0.01.  Certainty of Evidence: Moderate. The research followed a specific protocol and used statistical analysis, yet the absence of blinding and restrictions on sample size lower the overall confidence. | Repeated measures ANOVA was employed to examine fluctuation in MAS scores throughout the study period. Significant variations were observed in MAS scores for finger flexors, and subsequent tests indicated noticeable enhancements following tendon vibration (P<0.05). Statistical significance was attained using non-parametric tests such as the Kruskal-Wallis test and McNemar’s test for response rates. |

***Legend***: *Segmental muscle vibration (SMV), standard physical therapy (SPT), Barthel Index (BI), Activities of Daily Living (ADL), Modified Ashworth Scale (MAS), Manual Muscle Testing (MMT), range of motion (ROM), focal muscle vibration (FMV), progressive modular rebalancing (RMP), conventional physiotherapy (CP), Wolf Motor Function Test (WMFT), Visual Analog Scale (VAS), Motricity Index (MI), sensory-motor vibration (SMV), co-contraction index (CCI), Electromyographic (EMG), Fugl-Meyer Assessment Scale (FMAS), Modified Ashworth Scale (MAS), Maximum Voluntary Contraction (MVC), Biceps Brachii (BB), Functional Independence Measure (FIM), Jebsen-Taylor Hand Function Test (JTT), Verbal Numerical Rating Scale (VNRS), muscle vibration (MV), Short-interval intracortical inhibition (SICI), Hmax/Mmax ratio (HMR), Fugl-Meyer Assessment of Upper Extremity (FMA-UE), Hamilton Rating Scales for Depression and Anxiety (HRS-D, HRS-A), Mini-Mental State Examination (MMSE), Stair-Climb Test (SCT), Six-Minute Walk Test (6MWT), whole-body vibration (WBV), Serum cross-linked N-telopeptides of type I collagen (NTx), Sensory Organization Test (SOT), Functional Ambulation Classification (FAC), Targeted Rehabilitation Training (TRT), Fugl-Meyer Scale (FMS), medial gastrocnemius (MG), spinal cord injury (SCI), Neuropathic Pain Symptom Inventory (NPSI), Numeric Rating Scale (NRS), West Haven-Yale Multidimensional Pain Inventory: SCI version (MPI-PS), incomplete spinal cord injury (iSCI), American Spinal Injury Association Impairment Scale (AIS), 10-Meter Walk Test (10MWT), low-magnitude vibrations (LMV), transcranial magnetic stimulation (TMS), functional muscle vibration (FMV), non-invasive muscle vibration (NMV), right brain damage (RBD), left brain damage (LBD), Weight Bearing Asymmetry (WBA), Subjective Straight Ahead (SSA), Longitudinal Body Axis (LBA), Subjective Visual Vertical (SVV), Timed Up and Go Test (TUG), Berg Balance Scale (BBS), tibialis anterior (TA), biceps femoris (BF), Chedoke-McMaster Stroke Assessment (CMSA), Active range of motion (A-ROM), passive range of motion (P-ROM), vastus lateralis (VL), gastrocnemius (GS), Multiple Sclerosis (MS), Falls Efficacy Scale-International (FESI), Patient Determined Disease Steps (PDDS), Parkinson's disease (PD), Blood Pressure (BP), Systolic Blood Pressure (SBP), Diastolic Blood Pressure (DBP), Pulse Pressure (PP), Heart Rate (HR), Electrocardiogram (ECG), passive range of motion (PROM), Mini Balance Evaluation Systems Test (Mini-BESTest), Modified Modified Ashworth Scale (MMAS), Vibrotactile stimulation (VTS), Modified Tardieu Scale (MTS), Postural Assessment Scale for Stroke (PASS), Cerebral Palsy (CP), Drooling Impact Scale (DIS), Drooling Frequency and Severity Scale (DFSS), and Drooling Quotient (DQ), Intraclass Correlation Coefficient ICC).*
